# Color-Coding Method Reveals Enhancement of Stereotypic Locomotion by Phenazepam in Rat Open Field Test

**DOI:** 10.3390/brainsci13030408

**Published:** 2023-02-26

**Authors:** Mark Makarov, Yuri I. Sysoev, Oksana Agafonova, Veronika A. Prikhodko, Eduard Korkotian, Sergey V. Okovityi

**Affiliations:** 1 Faculty of Biology, Perm State University, 614068 Perm, Russia; 2 Department of Pharmacology and Clinical Pharmacology, Saint Petersburg State Chemical and Pharmaceutical University, 197022 Saint Petersburg, Russia; 3 Laboratory of Neuroprosthetics, Institute of Translational Biomedicine, Saint Petersburg State University, 199034 Saint Petersburg, Russia; 4 I.P. Pavlov Institute of Physiology of the Russian Academy of Sciences, 199034 Saint Petersburg, Russia; 5 N.P. Bechtereva Institute of the Human Brain of the Russian Academy of Sciences, 197376 Saint Petersburg, Russia; 6 Department of Brain Sciences, The Weizmann Institute of Science, Rehovot 7610001, Israel

**Keywords:** phenazepam, approaches in behavior study, stereotypic locomotion, dynamic patterns, behavior prediction, open field test, orientation disorder, planning skills impairments

## Abstract

One of the most important tasks in neuroscience is the search for theoretical foundations for the development of methods for diagnosing and treating neurological pathology, and for assessing the effect of pharmacological drugs on the nervous system. Specific behavioral changes associated with exposure to systemic influences have been invisible to the human eye for a long time. A similar pattern of changes is characteristic of phenazepam, a drug with a wide range of effects on the brain. In this study, we used a color-coding method, which consists of combining three time positions in one image, the present (0 s), the near future (0.33 s) and the far future (1.6 s). This method made it possible to identify movement patterns, such as the initialization of ahead movements, side turns and 180° turns (back), and also to determine the degree of predictability of future movements. The obtained data revealed a decrease in the number of turns to the sides while maintaining ahead movement, as well as an increase in the predictability of movements in rats under the influence of phenazepam. Thus, sedative doses of phenazepam do not exhibit general depression of brain functions, but the inhibition of specific centers, including the medial prefrontal cortex and postsubiculum, which are involved in stereotypic locomotive behavior.

## 1. Introduction

The study of behavioral patterns is one of the most difficult tasks in modern neuroscience. It requires the formation of a high level of qualification on the part of a human expert, painstaking continuous observation and a large investment of time. Fortunately, the use of automated analysis systems creates the conditions for significant facilitation and timesaving in research. Through the use of machine learning and the latest methods of analysis, it is possible to more deeply study the various behavioral patterns of groups of laboratory animals, such as rodents [1,2]. Moreover, prospects for the use of the developed methods in the diagnosis and treatment of various neuropathologies in humans are opening up [3,4,5,6]. 

For many years, animal behavior has been studied through visual observation, which has imposed certain restrictions on the research process, thereby affecting the data and results obtained during the analysis [7,8,9]. However, in recent decades, a trend has become noticeable for the mass introduction of computer technologies, machine learning, and various artificial intelligence systems. Thanks to the development of information technology, access to the latest methods of observation, recording and analysis of various kinds of data arrays has been created [10,11]. In particular, machine learning facilitates the comparison of different behavioral patterns, including the study of the dynamics of animal activity under different influences. Even simple image and video transformations can drastically change the conclusions about the behavior of the animal, expanding the possibilities of observation. This article has attempted to put some of these approaches into practice.

The development of approaches in diagnostics of various conditions, such as neurodegenerative diseases, latent viral infections, and side effects of neurotrophic drugs is a special class of tasks in neuroscience and behavioral neurobiology, in particular. These conditions affect the brain systemically and do not manifest themselves in an obvious and specific way at certain stages [12,13,14]. In this study, we chose rats medicated by phenazepam to model such a condition. Phenazepam is a well-known tranquilizer of the benzodiazepine group, with pronounced anticonvulsant, muscle relaxant and hypnotic effects [14,15,16,17]. The mechanism of action of phenazepam is to facilitate the inhibitory action of gamma-aminobutyric acid (GABA) on the transmission of action potentials in the central nervous system (CNS). In medical practice, phenazepam is prescribed for the treatment of nervous, neuropathic and psychopathic conditions accompanied by anxiety, fear and increased irritability [18]. The main neurological sites of involvement are primarily the amygdala and the reticular activating system [15], but GABA receptors are widely distributed throughout the CNS [19] and may be activated by phenazepam and associated with a wide range of effects, so we assumed that phenazepam is a good choice for our research. We were challenged to find some specific behavioral manifestations of phenazepam on the total background sedative effect, using observation and minimalistic computer transformations of data. Simplicity, easy accessibility and reproducibility of the applied methods were the conditions that we adhered to in the proposed study.

## 2. Materials and Methods

The series of experiments were carried out on 20 male Wistar rats (4 months old), weighing 250–300 g, obtained from Rappolovo Laboratory Animal Supplier (Leningrad Region, Russia). All behavioral tests were carried out in accordance with the principles of the Basel Declaration, the Order of the Ministry of Health and the recommendations of the institutional bioethical commission. In the home-cage vivarium, the standard temperature was maintained at 24 °C, the humidity level was set to standard. Rats received a balanced diet daily, maintaining a good level of locomotor activity. The light and dark cycle was set to 12/12 h. All ethical rules were considered, minimizing the level of stress and anxiety for the animals. 

All rats were selected from one batch and were quarantined for 14 days. Rats were divided into 2 groups according to the administered drug. Rats of the control group were injected with 0.5 mL saline. Phenazepam (Novosibkhimfarm), was administered as a solution of the same volume, at a concentration of 1 mg/kg [17].

During the study that was conducted with each of the two groups of rats, a number of behavioral parameters were analyzed in the open field test. The behavioral arena was a round field with a diameter of 97 cm. The field was divided into three rings, nested within each other (outer, intermediate and internal ones). The outer ring was additionally divided into 12 sectors, the intermediate one was divided into 6 sectors and the inner one was not divided. Thus, the arena was divided into 19 sectors of the same area, 389 cm^2^ each. In addition, the arena was equipped with 13 holes, evenly distributed on the floor.

Choosing the level of illumination in this work, we considered 2 factors. On the one hand, it was necessary to obtain a clear image, which is difficult without good lighting. On the other hand, it was important that the light did not cause excessive stress to the animal. The data obtained by Garcia et al. indicate that, in elevated plus maze, rat exploratory behavior occurring in the open arms (e.g., entries and time spent in these arms) was more intense under 0 and 1 lux than under the other illumination levels (3, 10, 30, 100 and 300 lux), which did not differ among themselves [20]. Based on this observation we chose the highest level of illumination, which is 320 lux. 

The exposure time after the administration of the drug was 20 min, after which the rat was placed in the arena of the experimental field. This waiting time was chosen in accordance with our previously published paper [17]. In this article, we observed that administration of phenazepam at the dose of 1 mg/kg cause pronounced EEG changes in rats 20 min after the injection.

After that, the experimenter left the room and filmed it for 3 min and 12 s. After each rat, the surface of the arena was cleaned with 95% ethanol and wiped with a clean paper towel. Before testing the next animal, the ethanol was allowed to evaporate completely.

The behavior was recorded using the VideoMot2 system (TSE Systems; Berlin, Germany). The camera viewpoint was located directly above the experimental arena. All video recordings were made between 12:00 and 15:00, adapted to the circadian rhythm of rodents. The recording camera was controlled remotely.

During the study, conducted with each of the two groups of laboratory rats, a number of behavioral parameters were analyzed, including mobility, anxiety and orienting-exploratory activity [8,21,22,23].

All 20 obtained records were processed in the Google Colaboratory service [24], using proprietary code. These video files included images consisting of three time positions that were expressed using color-coding. To obtain a color video recording, each individual video clip was divided into frames. Thus, three frames were used at each moment of time. Frame #1 represented the present time, frame #2 represented the near future (the 10th frame relative to the first one, i.e., in 0.33 s) and frame #3 represented the remote future (50th frame after the first one, i.e., in 1.66 s). Next, all frames were combined into a single video recording while maintaining the frame rate. The aim of color-coding was to determine the relationship of behavior patterns between the near and distant futures. As a result, if the relationship existed, it would be possible to predict individual patterns of behavior.

To classify the parameters of animal behavior, the following three criteria are usually considered:

1. Mobility;
2. Anxiety;
3. Orientation-exploratory activity.

The levels of mobility and anxiety were reflected in the number of intersections in different areas of the arena, such as “periphery” (12 outer sectors) and “center” (6 intermediate sectors + one internal). Thus, the total area of outer sectors was approximately 1.7 times greater than the internal ones. In addition, self-grooming is usually associated with over anxiety, in the sense that the more often grooming occurs, the higher the level of anxiety in rodents. Self-grooming was considered as body care, manifested as cleansing actions via the mouth and paws of the trunk, anogenital zone and tail, and care of the rostral part in the form of repeated movements of the front paws to the ears, head, face and nose [25,26]. Orientation-exploratory activity was represented by the frequency of rearing, the inspection of arena holes, rearing (standing up on the hind legs) with or without support on the wall of the field and air sniffing by patterns received by color-coding (number of ahead turn, side and back movements and the level of predictability of movements). The unit of measurement for these patterns is number of actions per record.

The data were statistically processed using Two-way ANOVA, Student’s *t*-test or Mann–Whitney U-test using PAST 4.03 and Excel 2016 (Microsoft; Redmond, WA, USA). Normality of data distribution in the samples was assessed using the Shapiro–Wilk test. Intergroup differences were considered statistically significant at *p* < 0.05. Data in bar charts are presented as M ± SEM.

## 3. Results

**Figure 1:**
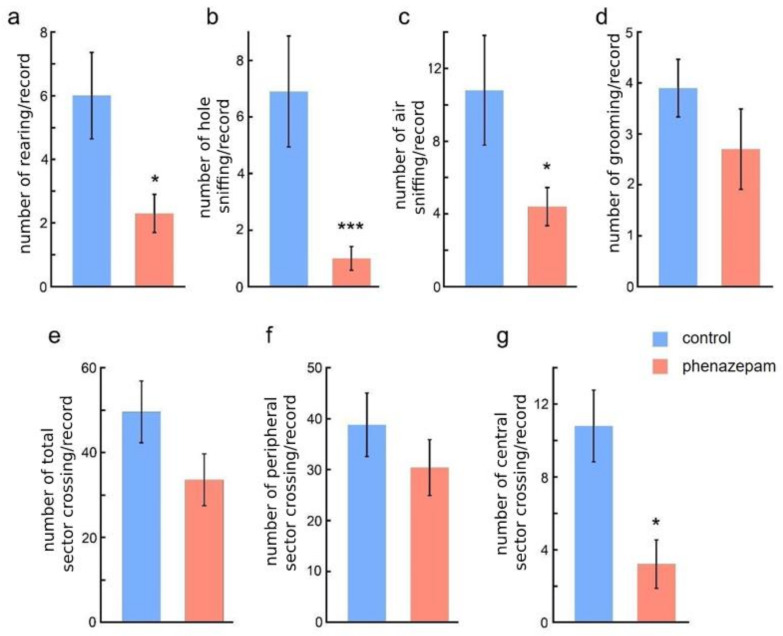
Effect of phenazepam on some rat behavior parameters in the open field test. Averaged exploratory behaviors (**a**–**c**), self-grooming (**d**) and locomotor activity (**e**,**f**) in the control (blue bars) and phenazepam (red bars) groups. (**a**) Averaged number of rearing with or without support on the wall of the field. (**b**) Number of arena hole sniffing episodes. (**c**) Air sniffing episodes. (**d**) Short or long grooming. (**e**) Overall crossing of arena sectors. (**f**) Only peripheral sector crossing. (**g**) Only central sector crossing. Units of measurement are number of episodes per record. *t*-test: *—*p* < 0.05; ***—*p* < 0.001; otherwise, not significant.

Initially, the effect of phenazepam on the behavior of rats in the open field was studied using standard approaches in behavior analysis. The results of these measurements are shown in Figure 1. 

Rearing with support on the wall of the field or without it is a manifestation of the orienting-exploratory reflex. Figure 1a shows that during three minutes of registration, control animals made about 6 rearing patterns, while following phenazepam, this number significantly decreased by more than two times. An even more pronounced difference was observed in the number of arena hole explorations. The frequency decreased by about 6 times, with a high level of statistical significance (Figure 1b). The number of air sniffing episodes, as another example of orienting behavior, was also reduced (Figure 1c). Wherein, the level of stress, indicated by grooming behavior, did not differ between the control and the phenazepam groups (Figure 1d). The same could be mentioned regarding the general mobility of animals based on the total number of crossed sectors and particularly, the intersections at the periphery of the field (Figure 1e,f). However, relocations among the central sectors, following phenazepam, decreased significantly, by about 3 times.

The presented data indicates that the general mobility, as well as the level of stress, were not pharmacologically affected, while exploration of the environment was significantly reduced.

Thus, the overall animal mobility following moderate doses of a tranquilizer does not change significantly and cannot serve as an indicator of the use of the drug. However, it was unclear whether the animal’s behavior in the open field test may reveal more subtle behavioral changes, such as planning, purposefulness and the precision of locomotion. To address this question, a relatively simple technique was developed to combine three temporal positions in one frame, including the current moment in time and the positions at 1/3 and 5/3 s. These time points were chosen for the following empirical reasons: in less than 1/3 of a second, the animal often did not yet move significantly, and in more than 1.7 s, the location could change too much so that the connection with the current position in the arena could be lost.

**Figure 2:**
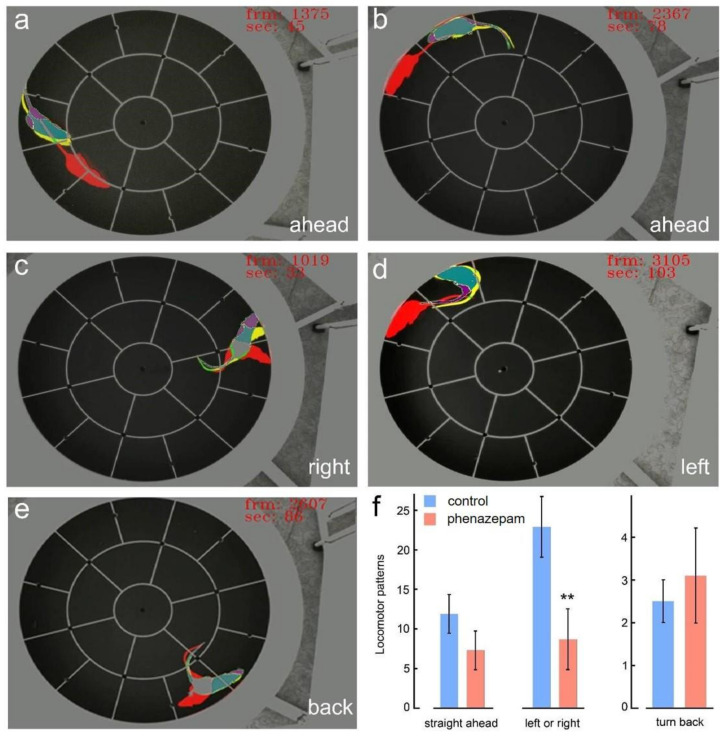
Temporal color-coding of rat locomotion in the open field test in control and phenazepam groups. Initial (present) rat position is represented in magenta, the “near future” position after 1/3 s shown in yellow, the overlap between the present and near future positions shown in dark green, the “far future” location after 5/3 s shown in red, the overlap between near and far future shown in light green, and the overlap of all three positions is shown in gray. Examples of basic locomotor behaviors are presented in panels (**a**–**e**) as follows: (**a**,**b**) movement straight ahead, (**c**,**d**) turns right and left, (**e**) turn back. (**f**) Averaged number of locomotor patterns in the control (blue bars) and phenazepam (red bars) groups as straight ahead, left, turns, middle, turn back, right pair of bars. *t*-test for the control/phenazepam pairs are as follows: straight, not significant; left or right, **—*p* < 0.03; back, not significant. Two-way ANOVA with control/phenazepam as factor 1 and the type of locomotion as factor 2 are as follows: factor 1—F = 20.27, *p* < 0.0001; factor 2—F = 2.08, *p* < 0.026; interaction—F = 1.13, *p* < 0.36 (MATLAB R2020a).

Based on the expert assessment and experience of the observer, three simple patterns of typical locomotor behavior were identified, that is, the position of the animal at 5/3 s relative to the present. In particular, forward movement, turns to the right/left (by about 90°) or back (by 180°) stood out. It should be noted that only unambiguous locomotor patterns were considered. For example, if the animal did not move strictly in a straight line ahead or moved along more complex trajectories than simple turns, then these cases were not considered. Examples of locomotor patterns are shown in Figure 2a,b for straight ahead, Figure 2c for the right, Figure 2d for the left and Figure 2e for the back turn.

As a result of a quantitative analysis of locomotor patterns, it turned out that the frequency of forward movements, although slightly reduced after phenazepam compared with the control, was not statistically significant. However, the difference in the number of turns to the left or right between the two groups was significant (*p* < 0.003 for the sum of turns in both directions, *p* < 0.0007 for left only and *p* < 0.03 for right only) (Figure 2f, left and middle bar graphs). The cases of turn back were relatively rare and approximately equal for both groups (Figure 2e,f, right bar graph). A two-way ANOVA test for the control/phenazepam groups as factor 1 and the types of locomotion as factor 2 revealed that both factors were significant (see Figure 2f caption), meaning that both the treatment and the type of locomotion affected the behavior, while interaction between the factors could not be observed. 

**Figure 3:**
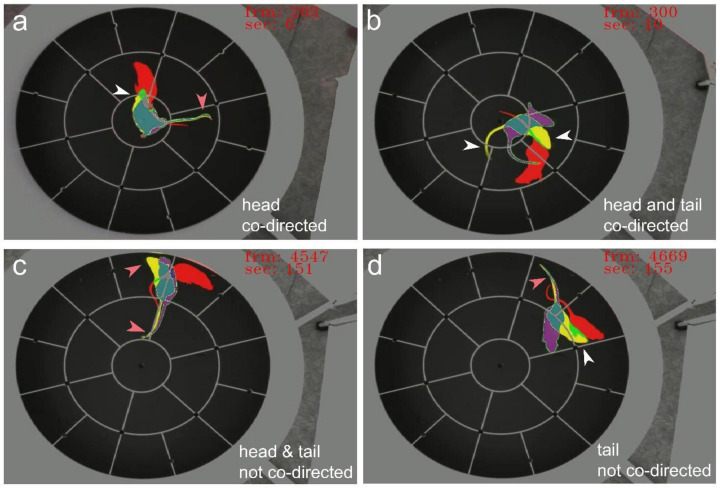
Examples of head and tail co-directional or non-co-directional positions regarding the future animal movement, revealed using temporal color-coding. For the time- and color-coding details, see the main text and the Figure 2 caption. (**a**) Head co-directed. (**b**) Both head and tail co-directed. (**c**) Both head and tail not co-directed. (**d**) Tail not co-directed. White arrowheads highlight co-directional positions and red arrowheads show non-co-directional positions.

This observation made it possible to analyze the position of the animal in the “near” future, that is, after 1/3 of a second, in the context of the position of the head and tail relative to the general direction of movement. The concept of a position “co-directed” to the distant future and a position not co-directed to it was introduced. So, if the head and/or tail of the animal were directed towards the future movement, with a deviation of no more than 30°, such a position was considered co-directional. In all other cases, it was considered not co-directional. Examples of these two head and tail positions are shown in Figure 3, with the white arrowhead pointing to co-directional movements and the red arrowhead marking non-co-directional or insufficient shifts. 

The number of locomotor behavioral patterns, as well as co-directional and non-co-directional positions of the head and tail of the animals varied quite pronouncedly from animal to animal, which made it difficult to average in absolute values. Therefore, percentage normalization was implemented, in which the total number of this pattern was taken as 100%. Relative to this value, the corresponding percentage of co-directional and non-co-directional head or tail displacements was calculated for each individual animal in each of the groups.

With such a presentation of the data, very significant and rather unexpected phenotypes were revealed. Turns to the left and right, as well as turns back were accompanied by co-directional head displacements in about 36–41% of the cases. Moreover, no statistical differences were observed between the control and phenazepam groups. The co-direction of these patterns with tail movements was observed somewhat less frequently, in 19–21% of cases, but here, too, both groups demonstrated statistically indistinguishable results.

**Figure 4:**
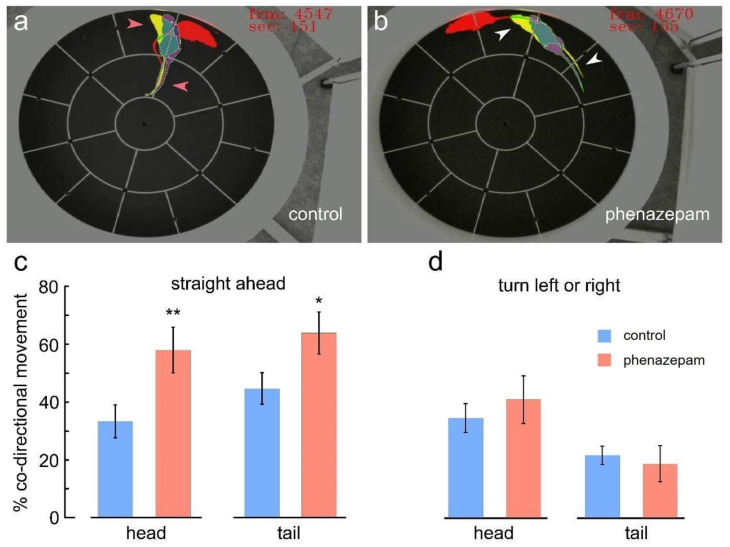
Differential percent of head and/or tail co-direction regarding the future rat locomotion. For the time- and color-coding details, see the main text and the Figure 2 caption. (**a**) Example of head and tail non-co-directional position in a control animal (red arrowheads). (**b**) Example of both head and tail co-directional positions in a phenazepam-treated animal (white arrowheads). (**c**) Averaged percent of head (left bars) and tail (right bars) co-directional positions in straight ahead locomotion for each recorded animal in the control (blue bars) and phenazepam (red bars) groups (*t*-test: *—*p* < 0.05; **—*p* < 0.01). (**d**) Averaged percent of head (left bars) and tail (right bars) co-directional positions in left/right turn locomotion for each recorded animal in the control (blue bars) and phenazepam (red bars) groups (*t*-test not significant).

When the animals moved straight ahead, the number of coincidences in the position of the head and tail was expectedly higher. However, if in the control the co-directivity was 46.9 ± 9.5 and 61.7 ± 7.1, respectively, then for phenazepam, the values were 80.5 ± 6.6 and 83.45 ± 4.6, respectively. These differences were clearly significant (*p* < 0.01 for the head and *p* < 0.02 for the tail). Typical locomotion phenotypes in the control and phenazepam groups are shown in Figure 4a,b. The corresponding averages are summarized in the bar graphs for control vs. phenazepam during forward movement pattern (panel c) and with left or right turns (panel d). These results suggest that tail and head positions are positive predictors of forward movements versus any turns for both groups. However, such a prediction works much more reliably with phenazepam rats compared to controls.

## 4. Discussion

The effect of phenazepam on the behavior of rats in the open field test was shown. Behavior change was assessed in the following three parameters: general mobility, anxiety, search behavior. The data indicate the effect of phenazepam on orienting and research activities in the absence of a significant effect on the state of stress, anxiety and general mobility. These conclusions are based on non-specific results of standard approaches (reduction in rearing, sniffing the air and holes, no difference in grooming and reduced visits to the central sectors by the phenazepam rats), on data obtained using color-coding and analysis of the trajectory of movement (changing the number of turns to the right and left while maintaining overall mobility) and on color-coding and co-directional movement analysis.

The anxiolytic effect of phenazepam is observed with the introduction of low doses of phenazepam (10^−10^ mol/kg) [27,28], but in our experiment, the level of anxiety in rats (at a dose of phenazepam of 1 mg/kg) remained unchanged. What could be the reason for such a dose-dependent effect? GABA_A_ receptors containing α_2_ subunits are associated with anxiety, schizophrenia and other cognitive diseases [29,30,31]. These receptors are more prevalent in the amygdala and hippocampus [19,32]. It is assumed that phenazepam molecules primarily bind to the GABA_A_ receptors of this zone, but higher doses of phenazepam lead to the inactivation of these receptors. Another assumption is that anxiety was assessed in different anxiety models and experiments were conducted on rats of a different breed and body weight. 

Is it possible to make conclusions about more specific reactions of orienting-exploratory behavior? The purpose of orienting-search behavior is a visual, tactile and olfactory study of the environment as a whole and its individual elements for further food search, reproduction and security [33,34]. All exploratory behavior of the rat is expressed in movements. For convenience we divide these movements into the following main types:

1. Movements associated with orientation in space;
2. Movements associated with the study of individual elements of space;
3. The beginning of movement and the movement itself.

The initiation of the movement and the movement towards the study area were registered in our experiments as forward movement, turns to the right or left and a back-turn. Such movements occur after the receipt of terrain data and the approval of the movement plan, irrelevant plans are not reproduced [35,36,37]. Then, the rat begins to move and in approximately half of the cases, after 0.33 s, its head is not directed towards the future movement, which also occurs in about 80% of cases for the tail. This set of movements can be interpreted in two ways. Firstly, movements may not be limited to moving from one point to another, rather they may be a multi-stage process that is planned even before the movement is initialized [37]. Secondly, the terrain data updates through the process of moving (even during the initialization of movement) to the planned area, therefore, the areas of interest for study are also updated. It may mean that there are prerequisites for the emergence of new routes that are more relevant than the one chosen before the moment the movement starts [38]. At this moment, a decision is made to change the plan, which explains the non-co-direction of the position of the head and tail at the beginning of the movement. 

In the phenazepam group, the co-direction of head movement in forward movement is noticeably higher than in the control group, which may be due to the inhibition of the centers responsible for the study of space, and centers (modules, connections) responsible for planning movements, such as lateral orbitofrontal cortex as a part of ventromedial prefrontal area, which receives inputs from the medial thalamus and probably takes part in emotional decision-making [37,39]. Therefore, following activation of the GABAergic system, movements or a movement plan may become simpler and more predictable than in the control group. Interestingly, this effect of phenazepam occurs only when rats are moving forward. The fact is that rectilinear movement is sufficiently provided by locomotor control from the extrapyramidal system, while any deviations from simple locomotion, including turns, require the participation of motor systems of a higher order [40]. In addition, it should be recalled that the frequency of turns to the side is reduced in rats from the phenazepam group, while the frequency of forward movements does not differ from control. It may be concluded that the number of side shifts is reduced due to the inhibition of higher control centers that are not associated with extrapyramidal locomotion, which is highly related to dopaminergic and serotonergic neurotransmission [41]. We hypothesize that “turn-deficiency phenomenon” may be explained by inhibition of postsubiculum (PoSub) head turning (HD) cells that modulate spatial information inputs from the antero-dorsal nuclei (ADv) of the thalamus [42], resulting in the rat being less oriented in space and continuing to explore the area along the wall as if the wall were long and did not end, which may explain the less frequent runs into the center by the phenazepam rats. A less trivial explanation is that the unmodulated signals from ADv orient the rat as if it was between two walls, so the head and tail are more often oriented in the forward direction.

Probably, the same cause of disorientation leads to a decrease in orienting movements, which include turning the head to the right and left, standing on the hind legs without support and sniffing the air. At the beginning of the experiment, these movements were made by the rat on the spot, and subsequently they were built into a series of movements, replacing each other. These movements are necessary for internalization of the map of the studied location, for entering information into the corresponding zones of the hippocampus [43,44] and for updating the security level. However, with the introduction of phenazepam, these movements become difficult to reproduce, since they require some orientation in space, which is disturbed due to the inhibition of HD cells.

The task of analyzing and diagnosing behavior when the brain is exposed to pharmacological drugs, viruses or neurodegenerative processes is very complex and requires ingenuity. In this study, we were able to demonstrate how, using fairly simple video transformations, and without the use of computer vision and artificial intelligence, we could reveal new patterns of behavior that can significantly enrich behavioral analysis. The structure of the obtained patterns made it possible to reveal completely new ones that were not previously known. In addition, the obtained data made it possible to put forward hypotheses about affected structures. This is useful for neurobiological research, as well as in research of new drugs. We believe that this work will contribute to research of the behavior of small, freely moving animals.

## Data Availability

Not applicable.

## References

[1] Andreev A., Ahremenko E., Apushkin D., Kuznetsov I., Kovalenko I., Korkotian E., Kalchenko V. New Approaches to Studying Rodent Behavior Using Deep Machine Learning. Proceedings of the International Conference on Advances in Digital Science.

[2] Godec P., Pančur M., Ilenič N., Čopar A., Stražar M., Erjavec A., Pretnar A., Demšar J., Starič A., Toplak M. (2019). Democratized image analytics by visual programming through integration of deep models and small-scale machine learning. Nat. Commun..

[3] Komura D., Ishikawa S. (2019). Machine learning approaches for pathologic diagnosis. Virchows Arch..

[4] Nichols J.A., Chan H.W.H., Baker M.A.B. (2018). Machine learning: Applications of artificial intelligence to imaging and diagnosis. Biophys. Rev..

[5] Thieme A., Belgrave D., Doherty G. (2020). Machine learning in mental health: A systematic review of the HCI literature to support the development of effective and implementable ML systems. ACM Trans. Comput.-Hum. Interact. (TOCHI).

[6] Bica I., Alaa A.M., Lambert C., van der Schaar M. (2020). From Real-World Patient Data to Individualized Treatment Effects Using Machine Learning: Current and Future Methods to Address Underlying Challenges. Clin. Pharmacol. Ther..

[7] File S.E. (1981). Animal tests of anxiety. Recent Advances in Neuropsycho-Pharmacology.

[8] Voikar V., Stanford S.C. (2022). The Open Field Test. Psychiatric Vulnerability, Mood, and Anxiety Disorders: Tests and Models in Mice and Rats.

[9] Acikgoz B., Dalkiran B., Dayi A. (2022). An overview of the currency and usefulness of behavioral tests used from past to present to assess anxiety, social behavior and depression in rats and mice. Behav. Process..

[10] Watson G.D.R., Hughes R.N., Petter E.A., Fallon I.P., Kim N., Severino F.P.U., Yin H.H. (2021). Thalamic projections to the subthalamic nucleus contribute to movement initiation and rescue of parkinsonian symptoms. Sci. Adv..

[11] Hsu Alexander I., Yttr Eric A. (2021). B-SOiD, an open-source unsupervised algorithm for identification and fast prediction of behaviors. Nat. Commun..

[12] Bellomo G., Piscopo P., Corbo M., Pupillo E., Stipa G., Beghi E., Vanacore N., Lacorte E. (2022). A systematic review on the risk of neurodegenerative diseases and neurocognitive disorders in professional and varsity athletes. Neurol. Sci..

[13] Calne D.B., McGeer E., Eisen A., Spencer P.S. (1986). Alzheimer’s disease, Parkinson’s disease, and motoneurone disease: Abiotropic interaction between ageing and environment?. Lancet.

[14] Nasrallah H.A., Chen A.T. (2017). Multiple neurotoxic effects of haloperidol resulting in neuronal death. Ann. Clin. Psychiatry.

[15] Cornett E.M., Novitch M.B., Brunk A.J., Davidson K.S., Menard B.L., Urman R.D., Kaye A.D. (2018). New benzodi-azepines for sedation. Best Pract. Res. Clin. Anaesthesiol..

[16] Manchester K.R., Lomas E.C., Waters L., Dempsey F.C., Maskell P.D. (2017). The emergence of new psychoactive substance (NPS) benzodiazepines: A review. Drug Test. Anal..

[17] Sysoev Y.I., Shits D.D., Puchik M.M., Prikhodko V.A., Idiyatullin R.D., Kotelnikova A.A., Okovityi S.V. (2022). Use of Naïve Bayes Classifier to Assess the Effects of Antipsychotic Agents on Brain Electrical Activity Parameters in Rats. J. Evol. Biochem. Physiol..

[18] Maskell P.D., De Paoli G., Seetohul L.N., Pounder D.J. (2012). Phenazepam: The drug that came in from the cold. J. Forensic Leg. Med..

[19] Herde A.M., Benke D., Ralvenius W.T., Mu L., Schibli R., Zeilhofer H.U., Krämer S.D. (2017). GABAA receptor subtypes in the mouse brain: Regional mapping and diazepam receptor occupancy by in vivo [18F]flumazenil PET. Neuroimage.

[20] Garcia A.M.B., Cardenas F.P., Morato S. (2005). Effect of different illumination levels on rat behavior in the elevated plus-maze. Physiol. Behav..

[21] Walsh R.N., Cummins R.A. (1976). The open-field test: A critical review. Psychol. Bull..

[22] Crusio W.E. (2001). Genetic dissection of mouse exploratory behaviour. Behav. Brain Res..

[23] Seibenhener M.L., Wooten M.C. (2015). Use of the open field maze to measure locomotor and anxiety-like behavior in mice. J. Vis. Exp. JoVE.

[24] (2022). Google Colab. https://colab.research.google.com.

[25] Estanislau C., Veloso A.W., Filgueiras G.B., Maio T.P., Dal-Cól M.L., Cunha D.C., Klein R., Carmona L.F., Fernández-Teruel A. (2019). Rat self-grooming and its relationships with anxiety, dearousal and perseveration: Evidence for a self-grooming trait. Physiol. Behav..

[26] Kalueff A.V., Stewart A.M., Song C., Berridge K., Graybiel A.M., Fentress J.C. (2015). Neurobiology of rodent self-grooming and its value for translational neuroscience. Nat. Rev. Neurosci..

[27] Voronina T.A., Molodavkin G.M., Chernyavskaya L.I., Seredenin S.B., Burlakova E.B. (1997). Effect of superlow doses of phenazepam on the EEG and behavior of rats in different models of anxiet. Bull. Exp. Biol. Med..

[28] Epstein O.I., Voronina T.A., Molodavkin G.M., Belopol’skaya M.V., Kheyfets I.A., Dugina J.L., Sergeeva S.A. (2007). Study of bipathic effect of phenazepam. Bull. Exp. Biol. Med..

[29] Heldt S.A., Ressler K.J. (2007). Training-induced changes in the expression of GABAA-associated genes in the amygdala after the acquisition and extinction of Pavlovian fear. Eur. J. Neurosci..

[30] Lewis D.A., Hashimoto T., Volk D.W. (2005). Cortical inhibitory neurons and schizophrenia. Nat. Rev. Neurosci..

[31] Rudolph U., Möhler H. (2014). GABAA receptor subtypes: Therapeutic potential in Down syndrome, affective disorders, schizophrenia, and autism. Annu. Rev. Pharmacol. Toxicol..

[32] Marowsky A., Fritschy J.M., Vogt K.E. (2004). Functional mapping of GABAA receptor subtypes in the amygdala. Eur. J. Neurosci..

[33] Mc Reynolds P. (1962). Exploratory behavior: A theoretical interpretation. Psychol. Rep..

[34] Hacques G., Komar J., Dicks M., Seifert L. (2021). Exploring to learn and learning to explore. Psychol. Res..

[35] Noonan M.P., Walton M.E., Behrens T.E.J., Sallet J., Buckley M.J., Rushworth M.F.S. (2010). Separate value comparison and learning mechanisms in macaque medial and lateral orbitofrontal cortex. Proc. Natl. Acad. Sci. USA.

[36] Noonan M.P., Chau B.K., Rushworth M.F., Fellows L.K. (2017). Contrasting Effects of Medial and Lateral Orbitofrontal Cortex Lesions on Credit Assignment and Decision-Making in Humans. J. Neurosci..

[37] Miller K.J., Venditto S.J.C. (2021). Multi-step planning in the brain. Curr. Opin. Behav. Sci..

[38] Goodroe S.C., Spiers H.J. (2022). Extending neural systems for navigation to hunting behavior. Curr. Opin. Neurobiol..

[39] Gremel C.M., Costa R.M. (2013). Orbitofrontal and striatal circuits dynamically encode the shift between goal-directed and habitual actions. Nat. Commun..

[40] Ben-Azu B., Aderibigbe A.O., Omogbiya I.A., Ajayi A.M., Iwalewa E.O. (2018). Morin pretreatment attenuates schizophrenia-like behaviors in experimental animal models. Drug Res..

[41] Yoshikawa S., Hareyama N., Ikeda K., Kurokawa T., Nakajima M., Nakao K., Mochizuki H., Ichinose H. (2009). Effects of TRK-820, a selective kappa opioid receptor agonist, on rat schizophrenia models. Eur. J. Pharmacol..

[42] Peyrache A., Schieferstein N., Buzsáki G. (2017). Transformation of the head-direction signal into a spatial code. Nat. Commun..

[43] Weiss S., Talhami G., Gofman-Regev X., Rapoport S., Eilam D., Derdikman D. (2017). Consistency of spatial represen-tations in rat entorhinal cortex predicts performance in a reorientation task. Curr. Biol..

[44] Jeffery K.J. (2018). The hippocampus: From memory, to map, to memory map. Trends Neurosci..

